# Effect of Peer Education on Knowledge of Human Papilloma Virus and Cervical Cancer among Female Adolescent Students in Benin City, Nigeria

**DOI:** 10.29024/aogh.24

**Published:** 2018-04-30

**Authors:** Ayebo Evawere Sadoh, Chukwunwendu Okonkwo, Damian Uchechukwu Nwaneri, Bamidele Charity Ogboghodo, Charles Eregie, Osawaru Oviawe, Omolara Famuyiwa

**Affiliations:** 1 Institute of Child Health, University of Benin, PMB 1154, Benin, NG; 2 Department of Obstetrics and Gynaecology, University of Benin, PMB 1154, Benin, NG

## Abstract

**Introduction:**

It is well documented that Human Papilloma Virus (HPV) is the cause of cervical cancer which is a major cause of morbidity and mortality especially in low- and middle-income countries. Vaccines against HPV are available. In developed countries where the vaccines have been deployed, lack of information among the target population (adolescents) is a major contributor to suboptimal uptake. In Nigeria, the vaccine is yet to be provided in the national programme on immunization, which is free, but it is available for a fee. In this study we determined the effect of peer education on the knowledge of female adolescents about HPV, cervical cancer, its treatment and prevention.

**Methods:**

This was an intervention study. The knowledge and awareness of female students of four secondary schools were assessed using a pre-tested self-administered questionnaire prior to the training of some of the students (peers). The trained students delivered messages on cervical cancer and HPV using fliers containing key information (peer training) to their school mates in formal delivery in a class setting. The knowledge and awareness of students, post-peer training, was then assessed.

**Results:**

There were 1337 students who responded to the baseline questionnaire while 1201 responded to the post-peer training questionnaire. Awareness of cervical cancer, knowledge of risk factors and cause of cervical cancer was low prior to the peer training. There was statistically significant improvement in awareness about cervical cancer and in the knowledge domains following peer training. Mean knowledge score prior to training was 12.94 ± 9.23 and this increased significantly to 53.74 ± 10.69 following peer training p < 0.0001.

**Conclusion:**

Peer training is effective in improving knowledge and awareness of secondary school students about HPV and cervical cancer.

## Introduction

Cervical cancer is the fourth most common cancer affecting women globally with low and middle income countries bearing a disproportionate burden of the disease [1]. Cervical cancer is the most common gynaecologic cancer in Nigeria [2]. The age-standardized incidence rate is in the range 20.6–30.2 per 100,000 population with mortality in the range of 9.8–17.0 per 100,000 population [1]. This makes cervical cancer a major cause of morbidity and mortality among women. The causative organism for cervical cancer is the Human Papilloma virus (HPV) with its over 100 serotypes [3]. However, vaccines against the HPV are available for use but the cost is prohibitive limiting its usefulness in the high-burden setting of low- and middle-income countries [4]. The vaccine is recommended for girls prior to their sexual debut. The HPV vaccine has not yet been deployed in the National Programme on Immunization (NPI) in Nigeria which provides vaccines free, but it is available for a fee. While the current high cost of the vaccine is a known barrier not only in Nigeria but also in developed countries [5], it is important to note that even the vaccines provided free in the National Programme on Immunization do not enjoy optimum coverage necessitating strategies that include mass mobilization and education as well as targeted advocacy based on identified barriers to immunization uptake [6].

Adolescence, the age group for which the vaccine is recommended, has peculiar characteristics which may be barriers to optimum uptake of the vaccine. It is an age when they tend to resist any dominant source of authority such as parents and they prefer to socialize more with friends than with family [7]. Research has also shown that they are more likely to modify their behaviour if they receive health messages from peers who face similar concerns and pressures [7]. It has also been documented that adolescents have problems when attempting to acquire information for their age-related concerns [8].

It is highly unlikely that cervical cancer will be discussed in a regular class setting in Nigeria, but it is important that adolescents acquire health-related information while at this stage of development as the information will prove useful before they take such decisions as commencement of sexual activity. Also, as many of these adolescents in Nigeria do not pursue higher education after secondary school [9], it is an opportunity to provide information that will be useful in their reproductive health when they become adults. More so, being the mothers of the next generation, they will be better equipped to take better health care decisions for their own children. It is also believed that the uptake of the vaccine will be improved if adolescents have information on the utility of the vaccine than if their parents simply request them to be vaccinated. Studies have, in fact shown that lack of information about cervical cancer and HPV vaccine is a major contributory factor to not being vaccinated [10,11].

One of the most important sources of reproductive health information for the adolescent is their peer. Several studies have shown the impact of peer education on improvement of knowledge [12,13]. Some studies have evaluated this approach in disseminating information on cervical cancer to adult women in Nigeria with good results [14,15]. In Nigeria, disseminating health information is challenging as trained and knowledgeable health educators are few and often not enough funds are provided for such activities. The aim of this study was to assess the knowledge of female secondary school students about cervical cancer and to determine if their knowledge could be improved using peer-to-peer transfer of knowledge.

## Methodology

**Study Locale**: Benin City is the capital of Edo state in the South-South geo-political zone of Nigeria. It is largely urban.

### Ethical consent

Ethical clearance for the study was obtained from the Ethical Review Committee of the College of Medical Sciences, University of Benin. Permission to carry out the study was obtained from the Principals of the various schools. Verbal consent/assent was obtained from the participating students.

### Study participants

Secondary education in Nigeria is provided by the government and private educational facilities. There are three levels in junior secondary (JSS1, JSS2 and JSS3) and three levels in the senior secondary (SS1, SS2 and SS3). Age at entry into secondary school is at least 10-years.

Female students from four secondary schools were recruited for the study. Baseline information was obtained from about 30% of the population of each school using a self-administered questionnaire. The questionnaires were distributed during break period and retrieved immediately after completion. The students were randomly selected. The baseline data was collected one week before a training seminar on Human Papilloma virus and cervical cancer was done.

Students from the different classes JSS1, JSS2, JSS3, SS1, SS2 and SS3 were selected to attend the training seminar. The lecture on cervical cancer and HPV was given by one of the authors (CO). Key information on cervical cancer was emphasized while each student was given a flier containing the key information. Within two weeks of the training, each student delivered a mini lecture on the subject to her classmates using the flier as a guide and to emphasize key points. At the seminar pre- and post-test assessment were done using the same questionnaire that was used for collecting baseline data.

Within a week of the students delivering their lectures, using the same questionnaire, information on cervical cancer and human papilloma virus was obtained from 30% of the students in each school. The questionnaires were also distributed during break period and retrieved immediately after completion. The pre-training questionnaires were not linked to post training questionnaires in the seminar and school cohorts.

### Study instrument

The questionnaire used for the pre- and post-test at the seminar as well as for collecting the baseline data in the schools and post-peer training in the schools was pretested at a secondary school that was not involved in the study. It consisted of two sections. The first section sought demographic information such as age, class, maternal and paternal levels of education and religion. The second section consisted of questions which sought information on awareness about cervical cancer and various knowledge domains such as causative organism, risk factors, treatment and prevention.

### Data analysis

Data was entered into an SPSS spread sheet and the same software was used for analysis. The students who responded to the questionnaires in school are referred to as the school cohort while those who received training at the seminar are called the seminar cohort.

The proportion of students with certain attributes was recorded as simple percentages. Association between variables was tested using Fishers Exact test and Chi-squared test as appropriate. Differences in variables between pre and post intervention were determined. Correct answers to questions on knowledge were awarded one point each while zero was awarded to non-response, wrong response or don’t know response. Total scores were converted to percentages. Mean knowledge scores were calculated for different categories of students. The significance of the difference between multiple means was tested using ANOVA. The level of significance was set at 0.05 at 95% confidence level.

## Results

There were 1337 female students in the school cohort from whom baseline data was collected. This consisted of 873 (65.3%) students from junior secondary and 420 (31.4%) from senior secondary school while 44 (3.3%) students did not indicate their classes. Following peer training in the schools, 1201 students consisting of 724 (60.3%) from junior secondary and 452 (37.6%) from senior secondary school responded to the post intervention questionnaire; 25 (2.1%) did not indicate their classes. The seminar cohort consisted of 124 students comprising 65 (52.4%) students from junior secondary school and 59 (47.6%) from senior secondary school. Table 1 shows the sociodemographic characteristics of the subjects. The median age of the students was 13-years with a range of 9 to 17 years.

**Table 1:** Sociodemographic characteristics of students in the seminar and school cohorts.

Characteristic	Seminar cohort		School cohort (Pre-intervention)		School cohort (Post-intervention)	

	n	%	n	%	n	%

**Mean age in years (SD)**	124	13.71 (1.71)	1337	13.01 (1.82)	1201	13.36 (1.64)
**Fathers LOE**						

Nil	3	2.4	50	3.7	36	3.0
Primary	23	18.5	196	14.7	222	18.5
Secondary	46	37.1	628	47.0	667	55.5
Tertiary	52	41.9	446	33.3	276	23.0
Not indicated	–	–	18	1.3	–	–
**Mothers LOE**						

Nil	0	0.0	72	5.4	9	0.8
Primary	29	23.4	208	15.6	285	23.7
Secondary	74	59.7	813	60.8	735	61.2
Tertiary	21	16.9	225	16.8	172	14.3
Not indicated	–	–	19	1.4	–	–
**Type of school**						

Junior secondary	65	52.4	873	67.5	724	61.6
Senior secondary	59	47.6	420	32.5	452	38.4
**Religion**						

Christian	122	98.4	1190	99.1	1214	90.8
Muslim	2	1.6	11	0.9	118	8.8
Not indicated	–	–	–	–	5	0.4

LOE-Level of education, SD-Standard deviation.

### Knowledge of cervical cancer

There was low awareness of cervical cancer among the students prior to the training with only 198/1337 (14.8%) and 18/124 (14.5%) of the school cohort and seminar cohort respectively having ever heard of cervical cancer Table 2. There was a statistically significant increase in awareness to 1174/1337 (97.8%) among the school cohort after the intervention p < 0.0001. Majority of the school cohort 940/1337 (70.3%) knew that cervical cancer was not uncommon in Nigeria which was not significantly different from 92/124 (74.2%) of the seminar cohort. Awareness about cervical cancer being common increased significantly following the intervention.

**Table 2:** Knowledge of students in the seminar and school cohorts about risk factors, treatment and prevention of HPV and cervical cancer.

Knowledge Domain	Respondents with correct response									
	Seminar Cohort					School Cohort				

	Pre n = 124%		Post n = 124 %		p value	Pre n = 1337%		Post n = 1201%		p value

**Awareness about CC**										
Ever heard of CC	18	14.5	120	96.8	< 0.0001	198	14.8	1174	97.8	< 0.0001
CC uncommon in Nigeria	92	74.2	113	91.1	< 0.0018	940	70.3	1101	91.7	< 0.0001
**Causative organism**										
Staph aureus	0	0.0	124	100.0	< 0.0001	68	5.1	1191	99.2	< 0.0001
Human Papilloma virus	0	0.0	124	100.0	< 0.0001	32	2.4	1192	99.3	< 0.0001
Gonorrhea	1	0.8	122	98.4	< 0.0001	37	2.8	1180	98.3	< 0.0001
Syphilis	0	0.0	124	100.0	< 0.0001	97	7.3	1191	99.2	< 0.0001
**Risk Factors**										
Multiple partners	0	0.0	109	87.9	< 0.0001	380	28.4	1101	91.7	< 0.0001
One sexual partner	0	0.0	108	87.1	< 0.0001	351	26.3	1093	91.0	< 0.0001
Presence of STI	0	0.0	124	100.0	< 0.0001	99	7.4	1181	98.3	< 0.0001
Bathe with hot water	0	0.0	94	75.8	< 0.0001	365	27.3	937	78.0	< 0.0001
**Susceptibility**										
Married women only	1	0.8	94	75.8	< 0.0001	122	9.1	910	75.8	< 0.0001
Young girls only	0	0.0	106	85.5	< 0.0001	362	27.1	1075	89.5	< 0.0001
Older women only	1	0.8	10	8.1	0.0100	186	13.9	984	81.9	< 0.0001
Those with children	0	0.0	117	94.4	< 0.0001	152	11.4	937	78.0	< 0.0001
**Treatment**										
Antibiotics	0	0.0	109	87.9	< 0.0001	90	6.7	1117	93.0	< 0.0001
Surgery	54	43.5	118	95.2	< 0.0001	505	37.8	1192	99.3	< 0.0001
Cold water bath	90	72.6	123	99.2	< 0.0001	622	46.5	1192	99.3	< 0.0001
Radiotherapy	43	34.7	74	59.7	0.0001	334	25.0	1099	91.5	< 0.0001
**Prevention**										
Abstinence	92	74.2	71	57.3	0.0073	621	46.5	616	51.3	0.1200
Pap smear	123	99.2	123	99.2	1.5020	585	43.8	1192	99.3	< 0.0001
Vaccination	123	99.2	123	99.2	1.5020	460	34.4	1192	99.3	< 0.0001
Better to treat than prevent	0	0.0	105	84.7	< 0.0001	437	32.7	1063	88.5	< 0.0001

Pre – Pre-training, Post – Post-training, CC – cervical cancer, STI – sexually transmitted infection, Staph – Staphylococcus.

Of the 124 students in the seminar cohort none knew the causative agent of cervical cancer which was not statistically different from the few 32/1337 (2.4%) of the school sample that knew that HPV was the causative organism p = 0.11. A few of the school sample ascribed other more commonly talked about organisms such as Staphylococcus aureus and syphilis as causative organisms. A statistically significant increase in knowledge of the causative organism increased to 100% and 99.9% in the seminar and school cohorts respectively following the training at the seminar and peer training in the schools.

### Knowledge of risk factors

None of those in the seminar cohort knew that cervical cancer was associated with multiple sexual partners and that cervical cancer could occur in anyone who had had sexual activity. None of them also knew that cervical cancer could occur in married women, older women, young girls and women who have had babies. The knowledge about the risk factors increased significantly following training at the seminar p < 0.0001. Some of the students in the school cohort were aware that cervical cancer could occur in those with multiple sexual partners [380/1337 (28.4%)] as well as in those with only one sexual partner [351/1337 (26.3%)] and also in married women [122/1337 (9.1%)], older women [186/1337 (13.9%)] young girls [362/1337 (27.1%)], and in those who have had babies [152/1337 (11.4%)]. However, the knowledge about these risk factors increased to 75.9–93.7% for the different risk factors and the increase in knowledge for each of the risk factors was highly statistically significant p < 0.0001 Table 2.

### Knowledge of prevention and treatment

The majority of those in the seminar cohort knew that cervical cancer could be prevented by regular PAP smear 123/124 (99.2%) and by vaccination 123/124 (99.2%). In contrast only 585/1337 (43.8%) and 460/1337 (34.4%) from the school cohort knew that yearly PAP smear and vaccination respectively were prevention strategies (p < 0.0001). Abstinence was considered a preventive strategy by 92/124 (74.2%) students in the seminar cohort and 621/1337 (46.5%) of the school cohort. The difference between the seminar and school cohorts with regards to abstinence as a preventive strategy was statistically significant p < 0.0001. There was a statistically significant improvement in the proportion of students in the seminar cohort who considered abstinence a preventive strategy following the training (p = 0.007) but the improvement following peer training in the school cohort was not statistically significant (p = 0.21).

Correct knowledge about treatment modalities among the seminar cohort was 54/124 (43.5%) for surgery and 43/124 (34.7%) for radiotherapy while it was 505/1337 (37.8%) and 334/1337 (25.0%) respectively for those in the school cohort. There was significant misconception that antibiotics was a treatment modality by 43/124 (34.4%) and 675/1293 (50.5%) among students in the seminar cohort and school cohort respectively. This misconception was corrected following training in both the seminar and school cohorts.

### Mean knowledge score

The mean knowledge score prior to training for the seminar cohort was 12.94 + 9.23 which was not significantly different from that of the school cohort 12.07 ± 9.74 Table 3. The mean knowledge scores were not significantly different between classes in the seminar cohort (p = 0.12) and the school cohort (p = 0.79). There was also no significant difference in the mean scores between different ages either in the seminar cohort (p = 0.78) or in the school cohort (p = 0.44).

**Table 3:** Pre- and Post-intervention mean knowledge scores of students in the seminar and school cohorts by class and age.

Variable	Seminar cohort						School cohort						

	n	Pre		Post		p value	n	Pre		n	Post		p value
		
		MKS	SD	MKS	SD			MKS	SD		MKS	SD	

Overall	124	12.94	9.23	60.39	9.75	< 0.0001	1319	12.07	9.74	1201	53.74	10.69	< 0.0001
**Class**													
JSS1	5	13.6	14.03	55.00	8.25	< 0.0012	297	11.57	10.00	37	53.08	11.76	< 0.0001
JSS2	19	17.68	11.65	59.16	9.00	< 0.0001	151	12.05	9.06	196	53.88	10.44	< 0.0001
JSS3	41	10.54	8.89	60.57	10.21	< 0.0001	425	12.15	9.53	483	53.28	10.84	< 0.0001
SS1	26	12.62	8.66	59.54	10.51	< 0.0001	176	11.70	9.66	165	55.59	10.63	< 0.0001
SS2	18	12.0	7.88	61.56	8.36	< 0.0001	135	12.41	10.91	174	53.15	10.44	< 0.0001
SS3	15	14.93	7.32	63.14	10.43	< 0.0001	109	13.10	10.24	111	53.37	10.90	< 0.0001
**Age**													
9	1	12.00	–	60.00	–	< 0.0001	2	4.00	5.66	3	56.00	14.42	< 0.0001
10	3	6.67	11.55	52.00	6.93	< 0.0001	125	10.85	8.89	49	55.67	10.03	< 0.0001
11	5	14.40	8.76	58.40	10.04	< 0.0001	129	12.99	11.08	75	53.92	10.69	< 0.0001
12	21	15.62	9.79	59.24	9.35	< 0.0001	299	11.49	9.05	216	52.95	10.34	< 0.0001
13	34	12.24	11.40	60.24	10.65	< 0.0001	319	12.29	9.82	378	53.59	10.95	< 0.0001
14	15	12.80	6.45	56.53	8.80	< 0.0001	134	11.49	9.45	157	53.10	10.81	< 0.0001
15	22	10.91	8.66	62.18	9.70	< 0.0001	144	12.86	10.82	173	54.26	10.21	< 0.0001
16	19	13.89	7.47	63.33	8.92	< 0.0001	141	12.82	10.13	106	55.25	11.13	< 0.0001
17	4	12.94	9.27	68.00	9.24	< 0.0001	26	11.85	9.01	34	52.59	11.41	< 0.0001

MKS – Mean knowledge Score, SD – standard deviation, JSS – Junior secondary school, SS – Senior Secondary.

Following training the mean knowledge score for the seminar cohort was 60.39 ± 9.75 which was highly statistically significantly different from the pre-training mean score (p < 0.0001). Following peer training the mean knowledge score of the school cohort 53.74 ± 10.69 was highly statistically significantly higher than their pre-training mean knowledge score p < 0.0001. The mean knowledge score for the seminar cohort post-training was statistically significantly higher than that of the school cohort (p < 0.0001). The mean knowledge scores were statistically significantly higher post-training for all classes and ages. However, there was no statistically significant difference in the mean scores between classes (p = 0.68) and between ages (p = 0.27) in seminar cohort following training. Following peer training there was no significant difference in the mean scores between classes (p = 0.26) and between different ages (p = 0.60) in the school cohort. The mean knowledge scores were also not significantly associated with maternal level of education in the pre-peer training school cohort (p = 0.11) and in the post-peer training school cohort (p = 0.63).

The mean knowledge score improved by 47.45 percentage points among those in the seminar cohort compared to 41.67 percentage points among those in the school cohort.

## Discussion

This study showed that majority of the students had never heard about cervical cancer even though most of them felt that it was not uncommon in Nigeria. This is in keeping with a systematic review which reported low awareness about cervical cancer in sub-Saharan Africa [16]. The finding in this study is, however, in contrast to findings in Hong Kong where up to 95.9% of school girls of similar age group to those in this study were reported to be aware of cervical cancer [17]. In this study, also in consonance with the low awareness about cervical cancer, more than 90% of the respondents did not know that HPV was the cause of cervical cancer. A few of the students attributed cervical cancer to more commonly known organisms.

Many studies [17,18,19,20] have reported a lack of knowledge about risk factors which is important in taking decisions to adopt healthy life-styles. This study also demonstrated sub-optimal knowledge about possible risk factors for acquisition of HPV and cervical cancer. Health education programmes for adolescents will need to emphasize these risk factors which are not only significant for HPV and cervical cancer but also for other sexually transmitted infections. This study also showed that the students did not appreciate the susceptibility of all females that were sexually active. This is in keeping with findings in previous studies among adult women in which most felt that they were not at risk for HPV and cervical cancer [14,21].

Sexual activity was low in the students studied as only about 3% had engaged in sex. The low prevalence of sexual activity in this study is in contrast to other studies from Nigeria and other developed countries [22,23,24]. The low sexual activity among these students provides a window of opportunity for providing them important sexuality information and health education prior to sexual debut. They are also at an opportune stage during which they can receive the HPV vaccine as majority are yet to commence sexual activity.

Interestingly, almost all the students in the seminar cohort knew about vaccination and PAP smear as prevention modalities for HPV and cervical cancer prior to the training. This was in contrast to the school cohort in which less than half of the students knew about these interventions. This suggests that the students who attended the seminar may have been told about this prior to their coming for the seminar. Lack of knowledge about preventive tools has been implicated in the low uptake of PAP smear and HPV vaccination [10,11,21,25].

There was marked improvement in most knowledge domains following training and peer training in the seminar and school cohorts respectively. However, there was no significant increase in the proportion of students who knew that abstinence was a preventive strategy for HPV and cervical cancer. It is not clear as to why this was so.

In this study there was no difference in mean knowledge scores between the different classes. This is contrast to findings in Hong Kong in which students in higher grades scored significantly higher in knowledge scores [17]. This difference may be due to the low availability of information in the general population. Studies from Nigeria have documented low knowledge in other population groups such as women living in a slum, market women and women attending antenatal clinics [21,26,27], although some studies have reported reasonable knowledge among others such as female health care workers and teachers [6,28]. With low general knowledge about cervical cancer in the population students are unlikely to have information about HPV and cervical cancer.

There was also no difference in the mean knowledge score between different ages. This is similar to findings in a Nigerian study on knowledge of secondary school students about cancer in general and cancer risk factors [22]. However, the finding was in contrast to findings from Europe in which knowledge was found to positively increase with age [29]. The general low information in the population and the tendency for a general avoidance of discussions on sexually related health information at family and community level may be responsible for the observations in this study.

In keeping with previous documentation on peer education this study showed that peer training was effective in improving knowledge in the study population. Peer education on cervical cancer has been successful in adult women but this study is the first, to the best knowledge of the researchers, to utilize this approach for disseminating information on HPV and cervical cancer to an adolescent population. Different methods have been proposed for the delivery of peer education [7]. The method used in this study was that of formal delivery in a class setting. The advantage of this method is the ability to increase knowledge in a large number of persons in a short period of time with minimal resources. The impact of this strategy is likely to be more as the adolescents were encouraged to discuss the information received with family members and with friends who are not in their schools. The use of key points to enhance recall and its use for peer training is an important method to emphasise important must-know facts.

This study has also shown that students at different stages of development (age and class) can be taught the same thing with good comprehension. There was no significant difference in mean knowledge scores of students who received similar training. Although mean knowledge score was significantly higher in the seminar cohort compared to the school cohort following the intervention, most knowledge domains were above 80%. The higher knowledge score in the seminar cohort could have been because they were assessed immediately after the training while for those in the school cohort assessment occurred within 3–5 days after peer training.

In conclusion, there was a dearth of knowledge about HPV and cervical cancer among female Nigerian adolescents in secondary schools. Peer education is an effective strategy for rapidly improving knowledge in the target group and portends optimal resource utilisation. It is recommended that all schools should use the strategy for disseminating information garnered from seminars outside their schools in which their students participated.
